# Surgery

**Published:** 1895-04

**Authors:** 


### SURGERY.

EDITED BY FLOYD W. MCRAE, M.D.

### SYMPTOMATOLOGY AND DIAGNOSIS IN SPINAL TRAUMATISMS WHICH INVOLVE THE OSSEOUS FRAMEWORK.

Abstract of an essay read before the Section on Surgery at the International Medical Congress at Rome, April 3, 1894, by Dr. T. H. Manley, of New York. Dr. Manley said: "The symptomatology of osseous disorganization in traumatisms of the spine is anything but definite; the bony segments of the vertebral column are so deeply lodged, so powerfully braced, and so shaped and heaped on each other by such an imbricated arrangement that nothing less than great and concentrated violence can cause a fracture of them, and when it does occur in the large majority of cases there is no visible or palpable displacement. In the greater number the cord entirely escapes, or only suffers such trival contusion that nothing more than a temporary numbness at the periphery, supplied by the nerve roots beyond the site of injury, follows. In the greater number of cases the apophyses alone suffer fracture when the transverse spinous process or posterior neck is involved; but it is only when the displacement of the fragments is inward and the cord is crushed through that symptoms of paralysis or paraplegia follow. There is every reason to believe that the spinal cord can bear moderate compression and laceration, combined with considerable longitudinal tension, without sustaining any serious damage. Experimentation has clearly proved that the spine may sustain fracture involving the apophyses and extend completely through the bodies without a single neural symptom following. In several animals on which I have instituted experiments to determine this question, I was able to produce the most extensive description of fracture, in several of which the spinous body was completely fractured horizontally across and through, thereby producing a distinct communication between the posterior mediastinum or pleural cavity and the spinal canal; and yet no paralysis of any kind succeeded the physical violence employed. Spinal fracture possesses no definite symptomatology; therefore, it may be said, its diagnosis in many cases is quite impossible.

"Fortunately for the patient, the failure to accurately locate and appreciate the precise extent and character of the fracture in no important manner influences treatment. Of late, since the advent of the antiseptic method of wound treatment, some have advocated the making of a free incision over the plane of suspected fracture, when we would be enabled to freely explore the suspected area, and, if need be, at the same time deal with the displaced fragments. At first sight this might seem a rational and prudent line of action, though if we investigate but superficially into the anatomic, structural composition of the spine, and consider what occurs in a fracture in this region, we shall the better appreciate its fallacious foundation. This spine is not, as has been alleged, in any manner analogous in structure or arrangement with the head; and, therefore, because we may generally, readily, and safely expose the brain, the same cannot be done with the spinal cord. The spinal cord, being very deeply lodged, is always very difficult of access and requires an extensive sacrifice of bone in order to make an opening of ample size to explore and manipulate through. And now, after our incision, which in this region is always attended with a great loss of blood where the bones are exposed: *a*, there may be no fracture; *b*, one or more fractures may escape detection; and *c*, if the fracture involves one or more of the bodies, our posterior incision will avail us nothing, as the cord, fixed in position by its nerve roots, obscures our view of their posterior surfaces. If, however, there has been a positive depression of the bony fragments on the cord and it is completely crushed through, of what avail will be an exploratory incision or a laminectomy in the way of repairing or restoring an organ totally destroyed beyond the point of compression? Not only is the diagnosis of spinal fracture often attended with quite insurmountable difficulties on the living, but even on the cadaver it may very often escape detection or be but imperfectly recognized by the *en courant* method of making necropsies on those who are presumed to have succumbed to spinal injuries, or they are associated with other internal complications. A post-mortem examination of the spine for a traumatic lesion of it, in order to be thorough, exact, and of any scientific value, always requires that the entire spinal column should be completely detached from the occiput, ribs, and sacrum. Then it should be brought into good light, and segment after segment, when the soft parts have been removed, be carefully manipulated and scrutinized. When the examination has been completed, it may be returned and fixed in the breach from which it was removed and be covered in."—*London Lancet.—Journal American Medical Association.*

---

### STRAPPING ULCERS.

Before a late meeting of the West-side German Dispensary, New York, Dr. Henry Roth said:

Strapping is applicable in the treatment of chronic ulcers resulting from varicose veins or parenchymatous dermatitis. It can be used in ambulant cases as well.

A green soap poultice is put on the ulcer and surrounding skin, with the object of cleansing the surfaces, at the same time applying a bandage from the toes to the knee. If, after removal of this, the ulcer is still dirty, antiseptic compresses can be used till the surface is rendered clean. Excessive granulations are cauterized with nitrate of silver, and systematic strapping may then be begun.

Surgeon's plaster is preferable to the ordinary adhesive plaster. The straps should be half to one inch in width and long enough to encircle two-thirds of the limb. Each strap must be heated over an alcohol flame before it can be applied.

First, we begin at the lower edge of the ulcer. One end of the first strap is fastened on healthy skin on one side of the ulcer, and then drawn firmly across to the other side, approximating the edges and encircling two-thirds of the leg. The next one is applied above the first and overlaps one-third of the lower strap. This is continued until the entire ulcer is covered.

Another method, which has been described by Dr. C. E. Quimby, is carried out in the following manner: The first strap bisects the ulcer; each succeeding strap bisects the remaining parts. This is continued until only strips of the ulcer, one-quarter to one-third of an inch in width are left uncovered. Then these are covered by narrower straps, which partially overlap those immediately above and below them.

The straps are removed in twenty-four to seventy-two hours and reapplied. In removing them both ends should be liberated at the same time, as this causes the least pain.

The advantages claimed for this method are several.

The margins of the ulcer are brought closer together, thus diminishing the surface to be covered by granulations.

By the pressure, which the straps exert, the everted and indurated margins are compressed, and soon are brought on a level with the base of the ulcer.

It improves the circulation and facilitates absorption. Excessive granulations are prevented by the firm pressure. Under this method improvement has been rapid.

---

### THE EFFECT OF ETHER ON THE KIDNEYS.

In the September number of the *University Medical Magazine* there is an article by Dr. George B. Wood entitled "The Elimination of Ether and its Relation to the Kidney," a thesis for which the Isaac Ott prize of the University of Pennsylvania for 1894 was awarded. The author gives accounts of seventeen experiments on animals, undertaken for the purpose of ascertaining the precise action of ether, when administered as an anesthetic, on the kidney, whether healthy or diseased. He thus summarizes the chief conclusions that he has arrived at: 1. It has been proved that ether exists as such in the free state in the blood, but, although it must come in close relation with the kidney, it is not excreted by that organ to any appreciable extent. Nevertheless it has been demonstrated that in ether anesthesia the kidney becomes congested, and on microscopical examination the cells show cloudy swelling. The cells of the convoluted tubules are affected primarily, and the tufts and collecting tubules do not show any change unless the anesthesia has been prolonged. It is probable that repeated administrations of ether, if kept up long enough, would cause desquamation of the epithelial cells. 2. The local effect of ether upon the kidneys already diseased must be very deleterious, for an unhealthy organ will not stand wear and tear like a normal one. In cases where uremic poisoning was beginning to manifest itself, it was shown that there was a liability to sudden death during ether anesthesia, due to the action of the ether on the already depressed centers of respiration.

The author gives it as his belief that in cases of nephritis surgeons should give ether only with the greatest care, and watch continually for any signs of failure of respiration. An important point, he says, is that the ether should be given very gradually, and when during the anesthetization it is necessary to use more ether, the inhaler should not be put directly on the face at once, but gradually brought close to it while the anesthetizer watches the patient's breathing carefully.—*Northwestern Lancet.*

